# Supplementary material for: High spin axion insulator
Source: Nat Commun. 2024 May 18;15:4250. doi: 10.1038/s41467-024-48542-4 (PMC11102527; doi:10.1038/s41467-024-48542-4)
Supplement: Supplementary file 1 — Supplementary Information [file 41467_2024_48542_MOESM1_ESM.pdf]

Supplementary Information for "High spin axion insulator"

Shuai Li[1,2], Ming Gong[3], Yu-Hang Li[4 ✉], Hua Jiang[5,6,2 ✉], and X. C. Xie[3,5,6,7]

[1]*School of Physical Science and Technology, Soochow University, Suzhou 215006, China*

[2]*Institute for Advanced Study, Soochow University, Suzhou 215006, China*

[3]*International Center for Quantum Materials, School of Physics, Peking University, Beijing 100871, China*

[4]*School of Physics, Nankai University, Tianjin 300071, China*

[5]*Institute for Nanoelectronic Devices and Quantum Computing, Fudan University, Shanghai 200433, China*

[6]*Interdisciplinary Center for Theoretical Physics and Information Sciences (ICTPIS), Fudan University, Shanghai 200433, China*

[7]*Hefei National Laboratory, Hefei 230088, China*

Contents

Note 1: Spin matrices for different spins 3

Note 2: Hamiltonian for high spin axion insulator from symmetry perspective 3

Note 3: Finite size effect 5

* E-mail: liyuhang@nankai.edu.cn, jianghuaphy@fudan.edu.cn

Note 4: Alternate detection **6**

Note 5: High spin axion insulator with spin-$\frac{5}{2}$ **7**

Note 6: Even-odd effect of the high spin axion insulator **14**

Note 1: Spin matrices for different spins

The spin matrices for different spin species can be generated mathematically from the Clifford algebra [1, 2], which are

spin-s	s_x	s_y	s_z
1/2	$\begin{pmatrix} 0, & 1 \\ 1, & 0 \end{pmatrix}$	$\begin{pmatrix} 0, & -i \\ i, & 0 \end{pmatrix}$	$\begin{pmatrix} 1, & 0 \\ 0, & -1 \end{pmatrix}$
3/2	$\begin{pmatrix} 0 & \frac{\sqrt{3}}{2} & 0 & 0 \\ \frac{\sqrt{3}}{2} & 0 & 1 & 0 \\ 0 & 1 & 0 & \frac{\sqrt{3}}{2} \\ 0 & 0 & \frac{\sqrt{3}}{2} & 0 \end{pmatrix}$	$\begin{pmatrix} 0 & -i\frac{\sqrt{3}}{2} & 0 & 0 \\ i\frac{\sqrt{3}}{2} & 0 & -i & 0 \\ 0 & i & 0 & -i\frac{\sqrt{3}}{2} \\ 0 & 0 & i\frac{\sqrt{3}}{2} & 0 \end{pmatrix}$	$\begin{pmatrix} \frac{3}{2} & 0 & 0 & 0 \\ 0 & \frac{1}{2} & 0 & 0 \\ 0 & 0 & -\frac{1}{2} & 0 \\ 0 & 0 & 0 & -\frac{3}{2} \end{pmatrix}$
5/2	$\begin{pmatrix} 0 & \frac{\sqrt{5}}{2} & 0 & 0 & 0 & 0 \\ \frac{\sqrt{5}}{2} & 0 & \sqrt{2} & 0 & 0 & 0 \\ 0 & \sqrt{2} & 0 & \frac{3}{2} & 0 & 0 \\ 0 & 0 & \frac{3}{2} & 0 & \sqrt{2} & 0 \\ 0 & 0 & 0 & \sqrt{2} & 0 & \frac{\sqrt{5}}{2} \\ 0 & 0 & 0 & 0 & \frac{\sqrt{5}}{2} & 0 \end{pmatrix}$	$\begin{pmatrix} 0 & -i\frac{\sqrt{5}}{2} & 0 & 0 & 0 & 0 \\ i\frac{\sqrt{5}}{2} & 0 & -i\sqrt{2} & 0 & 0 & 0 \\ 0 & i\sqrt{2} & 0 & -i\frac{3}{2} & 0 & 0 \\ 0 & 0 & i\frac{3}{2} & 0 & -i\sqrt{2} & 0 \\ 0 & 0 & 0 & i\sqrt{2} & 0 & -i\frac{\sqrt{5}}{2} \\ 0 & 0 & 0 & 0 & i\frac{\sqrt{5}}{2} & 0 \end{pmatrix}$	$\begin{pmatrix} \frac{5}{2} & 0 & 0 & 0 & 0 & 0 \\ 0 & \frac{3}{2} & 0 & 0 & 0 & 0 \\ 0 & 0 & \frac{1}{2} & 0 & 0 & 0 \\ 0 & 0 & 0 & -\frac{1}{2} & 0 & 0 \\ 0 & 0 & 0 & 0 & -\frac{3}{2} & 0 \\ 0 & 0 & 0 & 0 & 0 & -\frac{5}{2} \end{pmatrix}$

Supplementary Table 1: spin matrices for different spins.

Note 2: Hamiltonian for high spin axion insulator from symmetry perspective

The model Hamiltonian for the high spin axion insulator can be built from the high spin topological insulator preserving both parity and time-reversal symmetry [3]. These parity and time-reversal symmetry on the orbital and spin basis are defined as $\mathcal{P} = \tau_z$ and $\mathcal{T} = e^{-is_y}\mathcal{K}$, respectively, where τ_z is the Pauli matrix acting on orbitals, s_y is the spin matrix shown in Sec. Note 1:, and $\mathcal{K}$ is the complex conjugation operator. Since (the odd power of) the spin matrices $s_{x,y,z}$ flip sign under the time-reversal symmetry, the minimum Hamiltonian for a high-spin topological insulator preserving both parity and time-reversal symmetry thus

reads

$$H_0 = (m_0 - Bk^2)s_0 \otimes \tau_z + \sum_{i=x,y,z} A_i k_i s_i \otimes \tau_x, \tag{1}$$

where $k^2 = k_x^2 + k_y^2 + k_z^2$, m_0, B, $A_{i=x,y,z}$ are system parameters. The first term in Supplementary Eq. (1) is the kinetic energy while the second term represents the spin-orbital coupling. Due to the time-reversal symmetry, the axion field of H_0 is quantized. In the main text, we take the spin orbit coupling $A_x = A_y = A_z$ for convenience. However, our theory is universal and is not limited to special parameters. It also worths note that the spin orbital coupling is crucial for the quantized axion field, which otherwise becomes $\theta_0 = 0$ if $A_{i=x,y,z} = 0$. In the presence of magnetic ordering, the time-reversal symmetry is explicitly broken. However, in the antiferromagnetic phase, the combined lattice and time-reversal symmetry is still well preserved because the magnetic moments on the adjacent layers are antiparallel. The magnetic layers introduces an exchange interaction into the system, which recasts the Hamiltonian as

$$H = H_0 + \Delta \mathbf{m}_s \cdot \mathbf{s} \otimes \tau_0, \tag{2}$$

where Δ is the exchange gap between the magnetic moment $\mathbf{m}_s$ and the topological electrons with spin $\mathbf{s}$. In three dimension limit, the system preserves the symmetry $\mathcal{S} = T\tau_{1/2}$ with $\tau_{1/2}$ the half translation operator along z-direction. Whereas, this $\mathcal{S}$ symmetry breaks into combined parity and time-reversal symmetry that can be defined as $\mathcal{PT} = \sigma_z e^{-is_y}\mathcal{K}$ on a slab geometry, where σ_z is the Pauli matrix switching the magnetic moments between the top and bottom layers

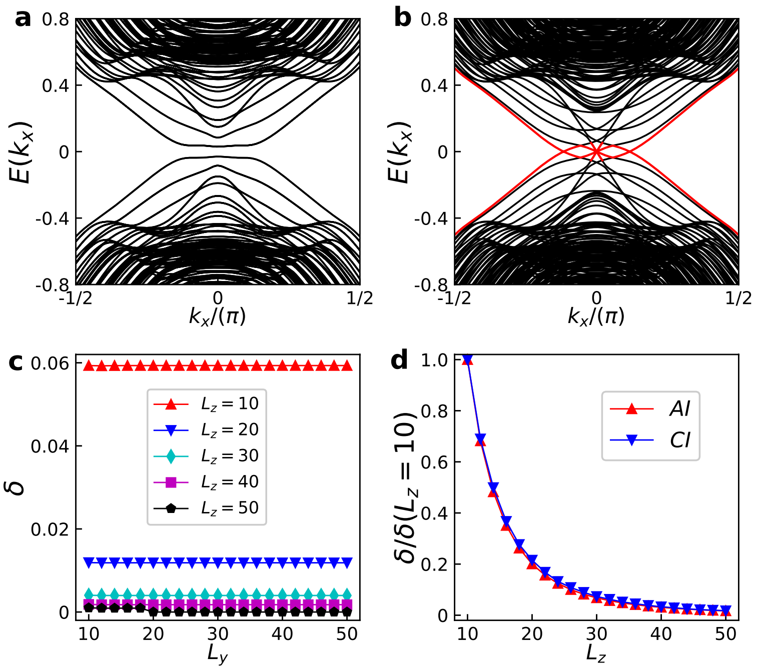

Supplementary Fig. 1: Size dependence of the energy gap. **a** and **b** show the band structures for systems in the antiferromagnetic and ferromagnetic cases, respectively. In the ferromagnetic case, the system is a spin-3/2 Chern insulator. The red lines in **b** denote the four gapless edge states. Here, the system size is $L_y = 20$, $L_z = 10$. **c**, energy gap for the HSAI versus L_y. **d**, size dependence of the HSAI band gap (red triangle) and Chern insulator bulk band gap (blue up-side-down triangle) with $L_y = 20$. The results for the Chern insulator bulk band gap is obtained after removing the surface states (red lines). All parameters for the model Hamiltonian are exactly the same as those in the main text.

Note 3: Finite size effect

To rule out the possibility of the finite-size effect induced energy gap in HSAI, we study the size dependence of the energy gap. Supplementary figures 1 a and b show the band structures of a one-dimensional nanowire with size $L_y = 20$ and $L_z = 10$ for the system in the antiferromagnetic and ferromagnetic cases, respectively. In the ferromagnetic case, the system is a spin-3/2 Chern insulator with a Chern number $C = 4$ (see Sec. Note 6:). Therefore, there are four gapless edge states denoted by the red lines in Supplementary Fig. 1 b, in sharp contrast to the gapped band structure for a HSAI in Supplementary Fig. 1 a. On the other hand, figure 1 c shows the band gap for the HSAI as a function of L_y. It is apparent that this gap is independent of the size L_y. To further rule out the possibility of

the finite-size effect along z-direction, we compare the band gap for the HSAI with the bulk band gap of the Chern insulator after removing the edge bands (red lines in Supplementary Fig. 1b). The results plotted in Supplementary Fig. 1d show that the two band gaps as functions of L_z coincide quantitatively with each other, which demonstrates that the band gap in HSAI is also induced by the bulk bands rather than the finite size effect due to the overlapping between edge states on top and bottom surfaces.

Note 4: Alternate detection

The temporal dependent asymmetric current output in the main text has the form

$$i(t) = \begin{cases} V_0 G_{31} \sin \omega_0 t, & 2n\pi/\omega_0 < t \leq (2n+1)\pi/\omega_0 \\ V_0 G_{13} \sin \omega_0 t, & (2n+1)\pi/\omega_0 < t \leq (2n+2)\pi/\omega_0 \end{cases} \tag{3}$$

for an arbitary integer n, where G_{ij} is the conductance from terminal j to terminal i as explained in the main text, V_0 and ω_0 are the amplitude and frequency of the alternative harmonic voltage input, respectively. Performing a Fourier transform with respect to t converts the current into the frequency domain, which yields

$$\begin{aligned} I(\omega) &= \frac{\omega_0}{2\pi} \int_{-\pi/\omega_0}^{\pi/\omega_0} dt e^{-i\omega t} i(t) \\ &= \sum_{n=-N/2}^{N/2} V_0 \left[G_{31} \int_{2n\pi/\omega_0}^{(2n+1)\pi/\omega_0} dt e^{-i\omega t} \sin \omega_0 t + G_{13} \int_{(2n+1)\pi/\omega_0}^{(2n+2)\pi/\omega_0} dt e^{-i\omega t} \sin \omega_0 t \right] \\ &= \sum_{n=-N/2}^{N/2} \frac{V_0 \omega_0 e^{-i2n\pi\omega/\omega_0}}{\omega_0^2 - \omega^2} (1 + e^{-i\pi\omega/\omega_0})(G_{13} e^{-i\pi\omega/\omega_0} - G_{31}). \end{aligned} \tag{4}$$

Therefore, the non-reciprocal conductance can be unveiled by the function $F(\omega) = |I(\omega)(\omega^2 - \omega_0^2)|/(2N\omega_0 V_0)$ with N the truncation of the summation n. If $\omega = 2\omega_0$, $F(\omega) = G_{13} - G_{31} = G_{13}^N$ with G_{13}^N the non-reciprocal conductance when N approaches infinity. However, the function $F(\omega) = 0$ if otherwise. Consequently, the quantized helical hinge current can be

readily detected by using the alternate method proposed in the main text.

Note 5: High spin axion insulator with spin-$\frac{5}{2}$

The spin matrices for spin-5/2 are 6×6 as shown in Sec. Note 1: because the magnetic quantum number m_z has six values ranging from $-5/2$ to $5/2$. As illustrated in Supplementary Fig. 2a, the model Hamiltonian for the high spin axion insulator (HSAI) defined on spin-5/2 space shares the same form as the spin-3/2 HSAI despite that the spin matrices are different. Thus, all quantities presented in the main text can be obtained identically by incorporating the spin matrices for spin-5/2. It is important to note that all parameters in this supplementary information are the same as those in the main text.

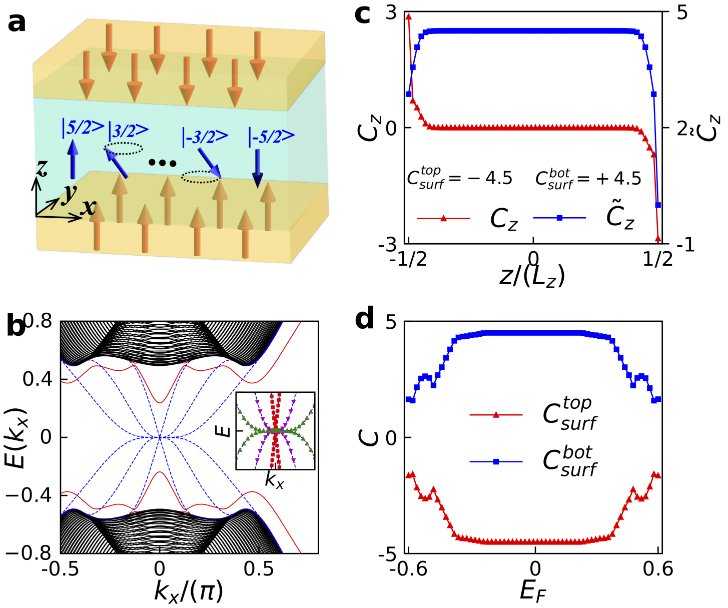

Supplementary Fig. 2: Model of the HSAI with spin-5/2**.** **a** Schematic for the HSAI with spin-5/2. The magnetic quantum number m_z takes values from $-5/2$ to $5/2$. **b** Two dimensional energy spectra for spin-5/2 HSAI along k_x ($k_y = 0$) in the absence (blue dashed lines) and presence (solid magenta lines) of the exchange interaction. The black lines are bulk bands. Inset: Solid lines are energy spectra of the HSAI without magnetic exchange term near the charge neutral point while markers are the fitting data. **c** Layer-resolved Chern number $C(z)$ and the cumulative Chern number $\tilde{C}(z) = \sum_{-1/2}^{z} C(z)$ versus the layer index z. **d** Surface Chern number as a function of the Fermi energy E_F. Here, the thickness of the spin-5/2 HSAI slab is $L_z = 60$ and the Fermi energy is $E_F = 0$.

Supplementary figure 2b shows the energy spectra for the spin-5/2 HSAI with and without the exchange term, respectively. In the absence of the exchange term, the system is a spin-5/2 topological insulator preserving both time-reversal and space inversion symmetries individually. It has a gaped bulk band but supports three gapless surface bands at each surface. In this case, the three surface bands can be perfectly fitted by a linear Dirac band $E_1 \sim k$, a cubic band $E_2 \sim k^3$ and a quintuple band $E_3 \sim k^5$ as shown in the inset in Supplementary Fig. 2b. The exchange term between the magnetic moments and the spin-5/2 topological electrons explicitly breaks the time-reversal symmetry of the HSAI and hence opens an exchange surface gap. When the Fermi energy lies inside the band gap, the lay-resolved Chern number $C(z)$ can be derived by projecting the TKNN formula into the specific layer. The results are shown in Supplementary Fig. 2c along with the cumulative Chern numbers $\tilde{C}(z) = \sum_{-L_z/2}^{z} C(z)$. We discover that the layer-resolved Chern numbers lie oppositely inside few surface layers, leading to a vanishing total Chern number $C = 0$. However, the surface Chern number in one side is $C_{bot(top)}^{surf} = \pm 9/2$, which indicates a distinctive axion term $\theta_{5/2} = (C_{bot}^{surf} - C_{top}^{surf})\pi = 9\pi$ [4]. This axion term is well preserved as long as the Fermi energy remains inside the band gap as shown in Supplementary Fig. 2d.

Even though the surface Chern number in spin-5/2 HSAI is not an integer, it still supports an identically quantized helical hinge current due to the difference of the quantum anomalous Hall conductances between top (or bottom) surface and the neighboring side surfaces. To explore this quantized helical hinge current, we examine the average position $\langle z/L_z \rangle$ (Supplementary Fig. 3b) as well as the energy spectrum $A(k_x, E)$ (Supplementary Fig. 3c) on a HSAI slab. The two results shown in Figs. 3b and c confirm a pair of helical hinge currents on the front surface of the HSAI slab at $y = -L_y/2$. Because of the inherent $\mathcal{PT}$ symmetry, two additional helical hinge currents exist on the other surface at $y = L_y/2$. The diagonal hinge currents are connected by this $\mathcal{PT}$ symmetry. They much propagate along the same direction. Therefore, in analogy to that in spin-3/2 HSAI and spin-1/2 axion insulator, the spin-5/2 HSAI also support helical hinge currents that propagate oppositely

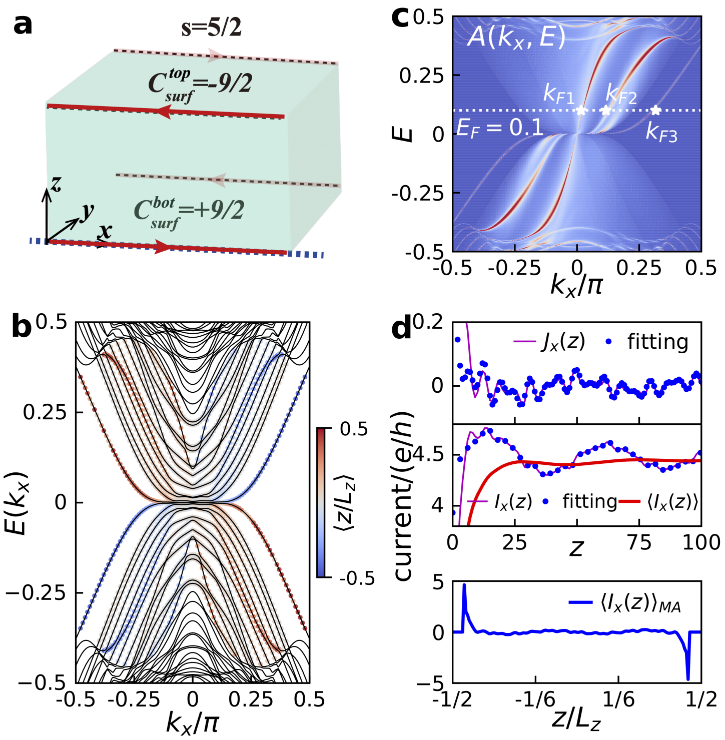

Supplementary Fig. 3: Quantized helical hinge current in HSAI with spin-$5/2$. **a** Schematic current flow in a spin-5/2 HSAI slab. **b** One dimensional energy spectrum and the average position $\langle z/L_z \rangle$ on the front surface as functions of k_x with $L_y = 20$, $L_z = 24$. **c** Spectrum density $A(k_x, E)$ for the front lower hinge as marked by the purple star in **a** on the $k_x - E$ plane. Here, the system size is $L_y = 20$, $L_z = \infty/2$. **d** Top and middle panels are the current density $J_x(z)$, current flux $I_x(z)$ and its z-averaged flux $\langle I_x(z) \rangle$ versus the layer index z for a semi-infinite system with size $L_y = 20$, $L_z = \infty/2$. The blue dots are the fitting data using three power law decaying edge currents with momenta k_{F1}, k_{F2} and k_{F3} as marked by the white dots in **c**. Bottom panel shows the distribution of the moving averaged current $\langle I_x(z) \rangle_{MA}$ on the front surface with system size $L_y = 20$, $L_z = 150$

on the neighboring hinges. To further quantitatively uncover those helical hinge currents, we calculate the current density $J_x(z)$, current flux $I_x(z)$, z-average current $\langle I_x(z) \rangle$ and the moving averaging current flux $\langle I_x(z) \rangle_{MA}$ in accordance with the formalism provided in the Methods. The results are plotted in Supplementary Fig. 3d. On one hand, $J_x(z)$ and $I_x(z)$ (solid magenta lines in the top and middle panels) coincide remarkably well with the fitting data (blue dots) obtained from the superposition of three power law decaying edge currents $J_x^{i=1,2,3} = a_i \cos(2k_{Fi}a_0 + \phi_i)/\sqrt{z}$, where k_{Fi} refers to the Fermi momenta marked by the white dots in Supplementary Fig. 3c, a_i and ϕ_0 are fitting parameters, certifing that the

beating mode of $J_x(z)$ comes from the superposition of the coherent edge currents on the same hinge. This beating mode occurs only in the HSAI since it originates from the superposition of two or more coherent edge currents while spin-1/2 axion insulator harbors only one edge current. On the other hand, one can see that $\langle I_x(z)\rangle$ and $\langle I_x(z)\rangle_{MA}$ quantize to $\pm 4.5e/h$ only a few layer away from the hinge. Thus, those signatures evidently confirms the quantized helical hinge current identical to the surface Chern number.

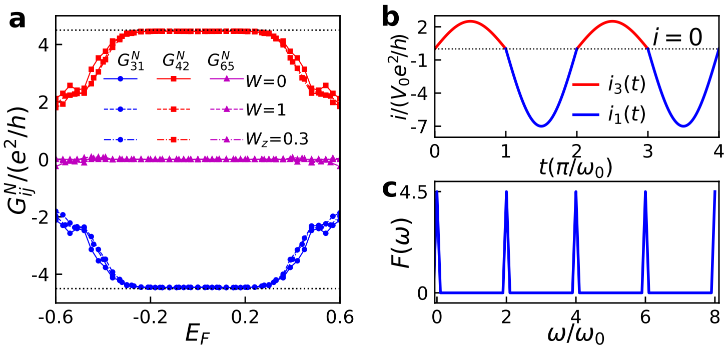

Supplementary Fig. 4: Transport properties of the spin-5/2 HSAI in a six terminal device. a Ensemble-averaged non-reciprocal conductances versus the Fermi energy in the clean limit, with non-magnetic Anderson disorders $W = 1$ and with magnetic Anderson disorders $W_z = 0.3$. Here, the system size is $L_x = 31$, $L_y = 20$, $L_z = 21$, and the size of transverse terminals is 10×10. **b** Corresponding temporal dependent current output between terminals 1 and 3 with parameters $G_{13} = 2.5e^2/h$ and $G_{31} = 7e^2/h$. **c** $F(\omega)$ as a function of the frequency ω.

In the same six terminal device as schematically shown in Fig. 2e in the main text, we calculate the non-reciprocal conductance for the spin-5/2 HSAI by using the non-equilibrium Green's function method. Three representative non-reciprocal conductances G^N_{31}, G^N_{42} and G^N_{65} as functions of the Fermi energy E_F are plotted in Supplementary Fig. 4a under different conditions (in the clean limit $W = 0$, with non-magnetic Anderson disorders $W = 1.0$ and with magnetic Anderson disorders $W_z = 0.3$). We see that $G^N_{31} = 4.5e^2/h$, $G^N_{42} = -4.5e^2/h$ and $G^N_{65} = 0$ when the Fermi energy lie inside the band gap regardless of the presence of disorders, confirming that those quantized non-reciprocal conductances are chiral and robust against both non-magnetic and magnetic disorders. As a result, the quantized helical hinge current is topological protected. This quantized helical hinge current can be

measured by using the alternate detection method, which eliminates the contribution from the conductive side surface. During the first half period, a positive voltage is applied to terminal 1, while current flows from terminal 3 is detected $[i_3(t)]$. Owing to the opposite chirality, the hinge current in this case is completely blocked, allowing only the current on the side surface to flow from terminal 1 to terminal 3. In the second half period, the harmonic voltage is moved to terminal 3, and the current output $i_1(t)$ from terminal 1 includes contributions from both the side surface and the hinge. Consequently, the temporal dependent current $i(t) = i_1(t) + i_3(t)$ is asymmetric as shown in Supplementary Fig. 4b. The amplitude difference between the positive and negative current originates from the chirality of quantized helical hinge current. This difference can be revealed quantitatively by the function $F(\omega)$ as explained in Sec. Note 4:, which equals to the non-reciprocal conductance $G_{13}^N = G_{13} - G_{31}$ when $\omega = 2\omega_0$ (Supplementary Fig. 4c). This further affirms that the quantized helical hinge current in HSAI with different spins can be experimentally observed via the alternate detection proposed in the main text.

We next examine the axion term and the topological magneto-electric effect bonded to it. The axion term can be calculated in terms of the hybrid Wannier functions on a two dimension HSAI slab with thickness L_z. Supplementary figure 5a shows the surface axion term θ_{CS}^{surf}, bulk axion term θ_{CS}^{bulk}, and the total axion term θ_{CS}^{slab} as functions of the inverse layer thickness $1/L_z$. It shows that the total axion term θ_{CS}^{slab} of the spin-5/2 HSAI is 9π when the system size approaches infinity while the bulk axion term quantizes to $\theta_{CS}^{bulk} = \pi$ simultaneously. Therefore, in analogy to the spin-1/2 case, the spin-5/2 HSAI is an axion insulator protected by the $\mathcal{PT}$ symmetry in the bulk. The surface axion term corresponding from the surface Wannier function is $\theta_{CS}^{surf} = 8\pi$, which is consistent with the Chern number of the surface Wannier function shown in the same figure and is independent of the system size. As the surface Wannier functions exist only on a slab geometry, they are independent of the system size and disappear under the periodic boundary condition. Thus, the axion term in the bulk for the spin-5/2 HSAI is π, which equals to θ_{CS}^{bulk} obtained here on a slab

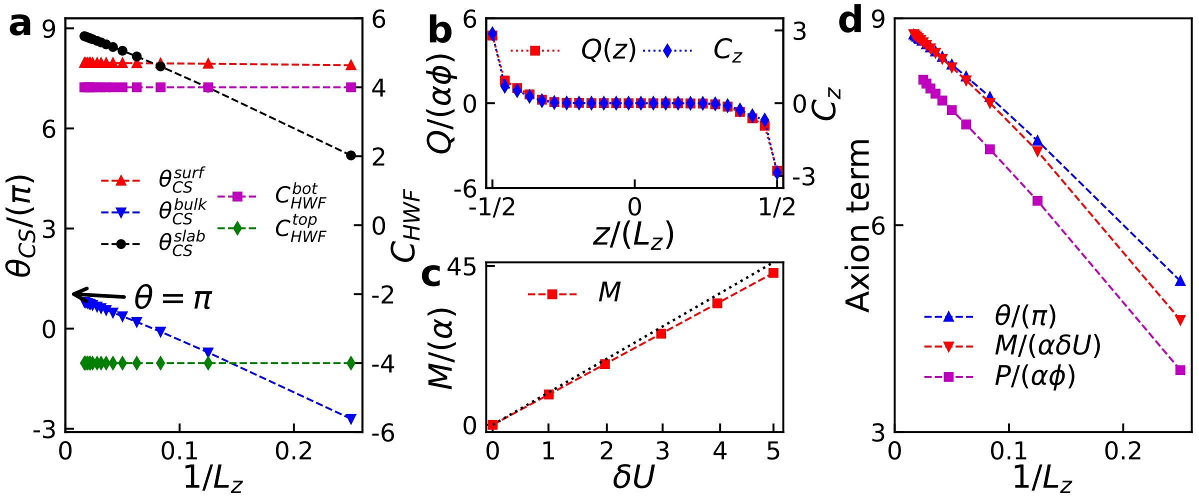

Supplementary Fig. 5: Axion term and the topological magneto-electric effect in HSAI with spin-5/2. **a** Axion terms and the surface Chern numbers versus the inverse layer thickness obtained by using the HWFs. **b** Magnetic field induced charge distribution along $\hat{z}$-direction and the layer-resolved Chern number for a spin-5/2 HSAI. Here, the charge polarization is obtained on a HSAI slab with open boundary condition along $\hat{y}$-direction ($L_y = 40$) but periodic boundary condition along $\hat{x}$-direction. **c** Electric field induced orbital magnetization for a spin-5/2 HSAI. The black dashed line shows the ideal case (IC) with an exact axion term $\theta = 9\pi$. **d** Size scaling of the axion term θ_{CS}^{slab}/π, polarization coefficient $P/(\alpha\phi)$, and magnetization coefficient $M/(\alpha\delta U)$. Here, the system size is $L_z = 24$.

geometry when the system size is infinity. This quantized axion term is also protected by the $\mathcal{PT}$ symmetry in analogy to the spin-1/2 axion insulator. The non-vanishing axion term of the slab indicates an identical topological magneto-electric effect. In the presence of an external magnetic field, the electrons can be pushed from one side to the other as plotted in Supplementary Fig. 5b, resulting in a charge polarization with the coefficient $P/(\alpha\phi)$ identical to the axion term θ_{CS}^{slab}, where P is the charge polarization, α is the fine structure constant and ϕ the total magnetic flux penetrating the HSAI slab. Furthermore, the charge distribution is consistent with the layer-resolved Chern numbers $C(z)$ obtained from the Bloch wave functions as shown in the same figure. By contrast, the electric field induced magnetization (red squares) shown in Supplementary Fig. 5c also agree quantitatively well with the ideal case with an exact quantized axion field $\theta_{IC} = 9\pi$ (black line), in which the slope of the data $M/(\alpha\delta U)$ refers to the magnetization coefficient with δU the total potential

drop across the HSAI slab. The slight deviation between them can be ascribed to the finite size effect, which is further unveiled by the size scalings of the axion term θ_{CS}^{slab}/π, polarization coefficient $P/(\alpha\phi)$, and magnetization coefficient $M/(\alpha\delta U)$ shown in Supplementary Fig. 5d. The results establish the equivalence of the slab axion term, the polarization coefficient and the magnetization coefficient.

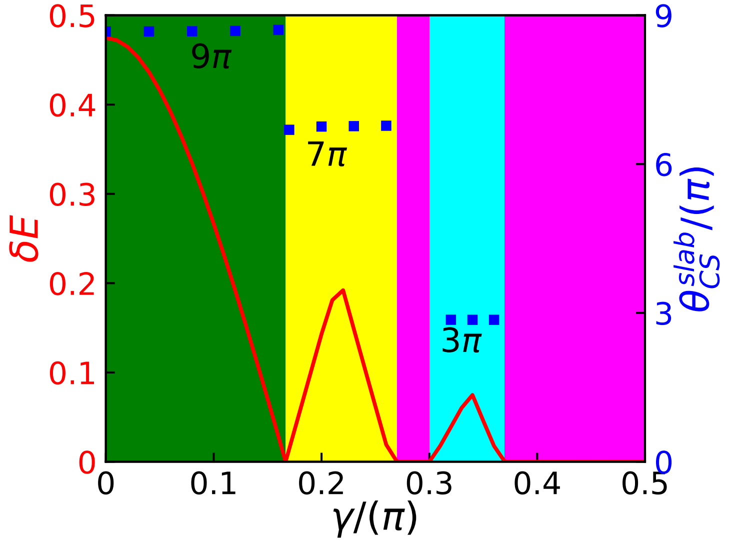

Supplementary Fig. 6: Topological phase transition in HSAI with spin-5/2. The red solid line plots the band gap for the HSAI with spin-5/2 as a function of the canting angle γ while the blue square refers to the corresponding axion term θ_{CS}^{slab}. The axion term is $\theta_{CS}^{slab} = 9\pi$ in the green region, $\theta_{CS}^{slab} = 7\pi$ in the yellow region, and $\theta_{CS}^{slab} = 3\pi$ in the cyan region. In the magenta regions, the gap is closed, therefore the axion term is not well-defined since the system is a metal. Here, the system size is $L_z = 40$.

Finally, we explore the topological phase transition in spin-5/2 HSAI under the driven of an in-plane magnetic field, which turns the antiparallel spins into canted spin state. The red line in Supplementary Fig. 6 plots the two dimensional energy band gap for the spin-5/2 HSAI as a function of the canting angle γ. It exhibits more gap closing and reopen than the spin-3/2 HSAI in the main text, which indicates much rich topological phase transitions. The blue squares in the same figure show the corresponding axion terms obtained using the hybrid Wannier function. We observe that the axion term changes from 9π (green region) to 7π (yellow region) then to 3π (cyan region) when the canting angle γ is enlarged. Moreover,

there exist two regions (magenta regions) where the band gap vanishes. In theses regions the system is a metal, therefore the axion term is not well-defined [5]. It is also important to note that the topological phase transition originates solely from the surface axion term θ_{CS}^{surf} while the bulk axion term θ_{CS}^{bulk} remains unchanged during the process. Because the transport signals in HSAI such as the non-reciprocal conductance identical to the quantized helical hinge currents and the topological magneto-electric response are proportional to the axion field, the HSAI thus provides a platform to realized the goal of axionic topological phase transition.

Note 6: Even-odd effect of the high spin axion insulator

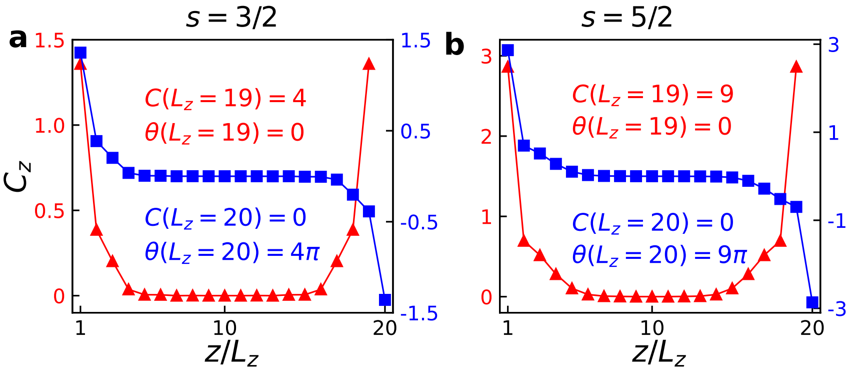

Supplementary Fig. 7: Even-odd effect of HSAI. a and **b** are layer-resolved Chern number for spin-3/2 and spin-5/2 HSAI with different layer thicknesses $L_z = 19$ (red triangle) and $L_z = 20$ (blue square). The Chern numbers and axion fields are labeled in the figure.

In the odd layer system, the net magnetization is non-vanishing because of the uncompensated magnetic layer. We can thus simulate this state by using parallel magnetic moments on both top and bottom layers. In order to reveal the difference between them, we explore the layer resolved Chern numbers, which can be obtained by using Eq. (4) in the methods. The results displayed in Supplementary Fig. 7 show that the layer-resolved Chern numbers for odd layer systems distribute symmetrically on the top and bottom layers, leading to a

vanishing axion field $\theta = 0$ while a nonzero Chern number $C = (s + 1/2)^2$ analogous to the odd layer $MnBi_2Te_4$ [6]. Therefore, the odd layer system is a high Chern number insulator. On the contrary, the even layer system is a HSAI with an axion field $\theta = (s + 1/2)^2\pi$ because of the asymmetric layer-resolved Chern numbers.

Supplementary References

[1] Lounesto, P. (2001). *Clifford Algebras and Spinors* (2nd ed., London Mathematical Society Lecture Note Series). Cambridge: Cambridge University Press.

[2] Lachieze-Rey, M., 2009. Spin and Clifford algebras, an introduction. *Advances in applied Clifford algebras*, **19**, pp.687-720.

[3] Rundong Li, Jing Wang, Xiao-Liang Qi, and Shou-Cheng Zhang, *Nat. Phys.* **6**, 284–288 (2010).

[4] Andrew M. Essin, Joel E. Moore, and David Vanderbilt, *Phys. Rev. Lett.* **102**, 146805 (2009).

[5] Xiao-Liang Qi, Taylor L. Hughes, and Shou-Cheng Zhang, *Phys. Rev. B* **78**, 195424 (2008).

[6] Lin, W., Feng, Y., Wang, Y. et al., *Nat. Commun.* **13**, 7714 (2022).
